# Non-Coding RNA Biomarkers in Prostate Cancer: Evidence Mapping and In Silico Characterization

**DOI:** 10.3390/life16010095

**Published:** 2026-01-08

**Authors:** Lorena Albarracín-Navas, Nicolás I. Lara-Salas, Javier H. Alarcon-Roa, Maylin Almonte-Becerril, Enmanuel Guerrero, Ángela L. Riffo-Campos

**Affiliations:** 1 Comprehensive Medical Services (SERMEDIC), Cuenca 010108, Ecuador; 2 Universidad de La Frontera, Ph.D. Program in Medical Sciences, Temuco 4780000, Chile; 3 Executive Direction of Research and Advanced Studies, Universidad de la Salud, Mexico City 01210, Mexico; 4 Faculty of Medicine, Universidad de Cuenca, Cuenca 010107, Ecuador; 5 SOLCA Cancer Institute, Cuenca 010105, Ecuador; 6 Center for Cancer Prevention and Control (CECAN), Santiago 8331150, Chile

**Keywords:** prostate cancer, non-coding RNA, miRNA, lncRNA, circRNA, snoRNA, piRNA, diagnostic biomarkers, in silico analysis, miRNA–protein interaction network

## Abstract

Non-coding RNAs (ncRNAs) have emerged as promising biomarkers for prostate cancer (PCa), yet evidence remains dispersed across heterogeneous studies and their regulatory context is seldom analyzed in an integrated manner. This study systematically maps ncRNAs reported as diagnostic biomarkers for PCa and characterizes their molecular interactions through in silico analyses. A comprehensive evidence-mapping strategy across major bibliographic databases identified 693 studies, of which 58 met eligibility criteria. Differentially expressed ncRNAs were extracted and classified by RNA type. Subsequently, miRNA–target prediction, miRNA–protein interaction network construction, and functional enrichment analyses were performed to explore the regulatory landscape of miRNA-associated proteins. Results: The final dataset included 4500 participants (2871 PCa cases and 2093 controls) and reported 94 differentially expressed miRNAs, eight lncRNAs, and several circRNAs, snoRNAs, snRNAs, and piRNAs. In silico analyses predicted 13,493 miRNA–mRNA interactions converging on 4916 unique target genes, with an additional 2481 prostate tissue-specific targets. The miRNA–protein network comprised 845 nodes and 2335 edges, revealing highly connected miRNAs (e.g., hsa-miR-16-5p, hsa-miR-20a-5p) and protein hubs (QKI, YOD1, TBL1XR1; prostate-specific CDK6, ACVR2B). Enrichment analysis showed strong overrepresentation of metabolic process-related GO terms and cancer-associated KEGG pathways. Conclusions: These findings refine the list of promising ncRNA biomarkers and highlight candidates for future clinical validation.

## 1. Introduction

Prostate cancer (PCa) represents a major global health challenge, ranking as the second most common malignancy among men. In 2022, an estimated 1,467,854 new cases were diagnosed worldwide, and PCa-related mortality reached 7.3% in men [1]. Projections indicate that global incidence will increase by 64.9% by 2045 [2]. This accelerating burden is driven largely by population aging, genetic susceptibility, and environmental factors [3]. Consequently, the need for diagnostic tools capable of accurately identifying clinically relevant tumors continues to grow. Although PSA testing and related clinical evaluations have facilitated earlier detection, these methods still struggle to reliably discriminate between indolent and aggressive disease, often resulting in unnecessary biopsies and overtreatment [4].

Advances in precision oncology have driven the exploration of new biomarkers that could complement, refine, or surpass traditional diagnostic strategies. Among these, non-coding RNAs (ncRNAs) have emerged as powerful regulators of gene expression, influencing pathways associated with proliferation, androgen signaling, genomic instability, and cellular plasticity [5]. A notable example is *PCA3*, one of the first lncRNAs to be incorporated into clinical practice as a urinary biomarker, through the PROGENSA^®^ *PCA3* Assay, which was approved by the U.S. Food and Drug Administration on 13 February 2012 [6,7].

Despite their potential, the landscape of ncRNA biomarkers reported for PCa remains fragmented. Studies frequently differ in their methodological approaches, nomenclature, definitions of biomarker positivity, and criteria for clinical utility [8,9]. Moreover, the functional interactions linking ncRNAs with their downstream targets, particularly miRNA–mRNA and miRNA–protein regulatory axes, are rarely examined in a comprehensive or comparative manner. This limits the integration of existing findings into biologically coherent models and hinders the identification of high-priority candidates suitable for clinical translation.

The present study addresses these gaps through a two-step strategy. First, we conducted a systematic mapping of all ncRNAs reported as diagnostic biomarkers for PCa, consolidating evidence from original research across multiple platforms and sample types. Second, we performed a series of in silico analyses, including miRNA–target prediction, miRNA–protein interaction network, and functional enrichment analysis, to characterize the regulatory landscape associated with ncRNAs currently linked to PCa. This work provides a comprehensive overview of ncRNA-based biomarkers and their molecular context, offering new insights into their diagnostic potential and guiding future validation efforts.

## 2. Materials and Methods

### 2.1. Literature-Driven Biomarker Selection

This literature search was conducted following the recommendations proposed by the Preferred Reporting Items for Systematic Reviews and Meta-Analysis (PRISMA 2020) statement [10] and the methodology was the same used for Albarracín-Navas et al. 2024 [11].

#### 2.1.1. Eligibility Criteria

The inclusion criteria were as follows: studies involving adult male populations (≥18 years) with prostate cancer that included both case and control samples and reported non-coding RNAs (ncRNAs) showing differential expression associated with disease diagnosis. For studies presenting transcriptomic data, only those that included an independent validation cohort were considered eligible, thereby prioritizing biomarkers with greater translational relevance and proximity to potential clinical implementation. The exclusion criteria included in vivo, in vitro, and in silico studies that lacked an independent validation cohort, in order to minimize false-positive findings and reduce bias associated with exploratory or discovery-only analyses; Gray literature, systematic and narrative reviews, short communications, opinion articles, letters to the editor, conference abstracts and proceedings, consensus statements, clinical trial protocols, case series, and case reports were excluded; Method studies and bioinformatics tools were also excluded; Articles on patients with metastases, or treatment interventions; Differentially expressed genes related to risk, progression, recurrence; Protein-coding genes were included in a previous study, thus excluded in this review. When the full text of the study was not available online, attempts were made to contact the corresponding author and libraries, and when this was not possible, the study was excluded. The selected primary articles were published up to 24 September 2023.

#### 2.1.2. Information Sources and Search Strategy

The systematic search was conducted across the bibliographic databases MEDLINE (via PubMed), Scopus, Embase, and Web of Science. In addition, a broader search strategy was applied by removing the term diagnosis, and this approach, together with a manual screening of the reference lists of the selected articles, enabled the identification of relevant studies not captured in the initial search. The search strategy combined free-text terms and controlled vocabulary (MeSH and DeSH), using the Boolean operators AND, OR, and NOT (see Supplementary File Sheet S1. Search Strategy).

#### 2.1.3. Selection of Articles

Authors (L.A.-N. and Á.L.R.-C.) conducted an independent, blinded, and systematic search of the bibliographic databases. Any discrepancies identified during this stage were resolved by consensus. Duplicate records were identified and removed (see Supplementary File Sheet S2). Subsequently, two reviewers (L.A.-N. and E.G.) independently screened titles and abstracts according to the predefined inclusion criteria, excluding studies that did not meet at least one criterion. In cases of disagreement, a third reviewer (Á.L.R.-C.) adjudicated and determined the final eligibility status. Finally, two reviewers (L.A.-N. and M.A.-B.) independently assessed the full texts of the remaining articles, and any disagreements were resolved by a third reviewer (Á.L.R.-C.), who issued the final decision.

#### 2.1.4. Data Collection Process

A standardized data extraction form was created using Microsoft Excel (version 15.24; Microsoft Corporation, Redmond, WA, USA, 2016), and all relevant variables from the included studies were systematically recorded (see Supplementary File Sheet S6).

The extracted variables included study design, gene symbol, reported *p*-values, fold change, statistical methods, RNA analysis techniques, biological sample type, and surgical treatment performed on participants. In addition, a structured summary was compiled for each article, capturing the digital object identifier (DOI), authors, year of publication, country of origin, population characteristics (number of cases, controls, and total participants), and age. The information was retrieved independently by three reviewers (L.A.-N., M.A.-B., and Á.L.R.-C.) and differences were resolved by two other reviewers (J.H.A.-R., and N.I.L.-S.).

#### 2.1.5. Risk of Bias in Individual Studies

Two independent researchers (J.H.A.-R. and N.I.L.-S.) assessed the methodological quality of the included studies using the Standard Quality Assessment Criteria for Evaluating Primary Research Papers from a Variety of Fields (QualSyst), specifically the checklist for quantitative studies [12]. This tool was selected due to its flexibility and suitability for evaluating heterogeneous study designs, which is a key characteristic of the ncRNA biomarker literature included in this evidence-mapping study. Each article was evaluated according to predefined methodological criteria, and a compliance percentage was calculated as the ratio between the score obtained and the maximum possible score. Scores of these tools range from 0, indicating the lowest methodological quality, to 1, indicating the highest level of methodological rigor.

### 2.2. In Silico Analysis

#### 2.2.1. miRNA Target Predictions

The miRNA obtained in the primary studies were searched in the Gene database from NCBI [13] for the official gene symbol identification (Supplementary File Sheet S7). Some primary studies did not identify the 5p or 3p miRNA origin; thus, the information was searched in the Supplementary Data or deduced from the primers used in the Section 2.

The web-based tools miRDB [14], TargetScan v8.0 [15] and miRTarBase v9.0 [16] were selected to integrate complementary computational and experimentally validated evidence. miRDB and TargetScan provide sequence- and conservation-based predictions, whereas miRTarBase includes experimentally supported miRNA–target interactions. (see Supplementary File Sheet S8). To ensure high-confidence results, only miRNA–mRNA interactions consistently identified across all three databases were retained for downstream analyses. This conservative, intersection-based approach prioritizes specificity and robustness, minimizing false-positive associations and enhancing interpretability in the context of biologically heterogeneous prostate cancer datasets. The filter Tissue Prostate was used in miRTarBase for the identification of tissue-specific targets (see Supplementary File Sheet S9). The TargetScan (http://www.targetscan.org/vert_72/); miRDB (http://mirdb.org/); and miRTarBase (https://mirtarbase.cuhk.edu.cn/), were accessed on 21 June 2025.

#### 2.2.2. The miRNA–mRNA Target Interaction Network Analysis

The miRNAs and their corresponding target genes were analyzed using the STRING database [17]. STRING assigns a confidence score ranging from 0 to 1, which reflects the likelihood that a given protein–protein interaction is biologically meaningful, specific, and reproducible. In this case, a confidence score of 0.9 or greater was included, selecting the interaction type experimentally determined. These parameters were chosen to ensure that the resulting interaction networks represent biologically meaningful and reproducible relationships. Although lower confidence thresholds can increase network coverage and sensitivity, they also introduce a higher proportion of predicted or indirect interactions, which may obscure core regulatory structures. Accordingly, our approach prioritizes specificity and interpretability, allowing for a more robust identification of central hubs, key protein targets, and significantly enriched pathways with established biological relevance. The network was analyzed and displayed using the yFiles organic layout with Cytoscape v3.9.1 [18].

#### 2.2.3. Network Enrichment Analysis

Enrichment analyses were performed using the STRING v12 platform [17], incorporating available information on relationships between network nodes based on Gene Ontology (GO) categories, including Biological Process, Molecular Function, and Cellular Component [19], as well as KEGG pathway annotations [20]. These annotations were integrated directly through the STRING framework to characterize the biological processes and pathways represented in the interaction network.

## 3. Results

### 3.1. Evidence Mapping

**Figure 1:**
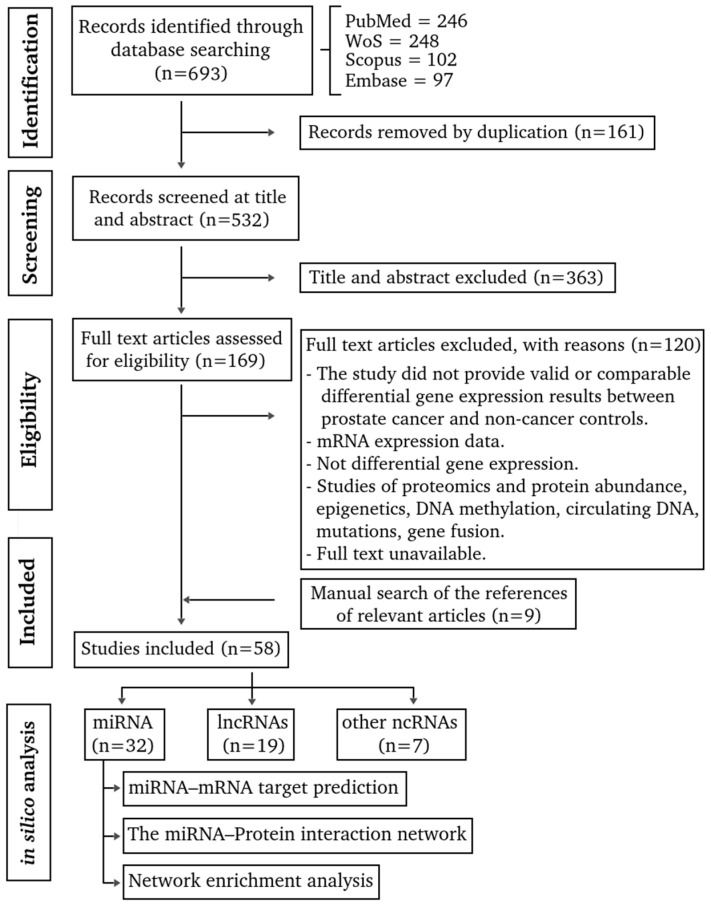
Flow diagram of the selection process and exclusion criteria for primary studies in the literature-driven identification of ncRNA biomarkers validated in prostate cancer patients. References for 58 primary articles were included in the Supplementary Materials.

A total of 693 articles were identified (Figure 1, Supplementary File Sheet S2). Of these, 161 duplicate articles were excluded. The other 363 articles did not meet the eligibility criteria during the screening of titles and abstracts (Supplementary File Sheet S3). The remaining 169 articles were analyzed by reading the full text, and 120 did not meet the eligibility criteria and were excluded (Supplementary File Sheet S4). Nine relevant articles were added from the manual search (Supplementary File Sheet S5), which raised the final count to 58 articles (Supplementary File Sheet S6) [21,22,23,24,25,26,27,28,29,30,31,32,33,34,35,36,37,38,39,40,41,42,43,44,45,46,47,48,49,50,51,52,53,54,55,56,57,58,59,60,61,62,63,64,65,66,67,68,69,70,71,72,73,74,75,76,77,78,79].

Most of the articles did not report their study design (*n* = 54); the remaining were described as pilot (*n* = 2), case–control (*n* = 1), and retrospective (*n* = 1) studies. Although the majority did not specify an experimental design, they included both tumor and control samples. Most studies originated from Asia (*n* = 25) and Europe (*n* = 22), followed by North America (*n* = 7), South America (*n* = 2), and Africa (*n* = 2). The dataset comprised 4500 participants (mean age = 65.0 ± 7.18 years), including 2871 case samples (57.8%) and 2093 control samples (42.1%). In certain studies, paired tumor and adjacent non-tumor tissues from the same patient were analyzed. Reported treatments comprised radical prostatectomy (*n* = 21) and simple prostatectomy (*n* = 4); in 33 studies, treatment information was not provided.

The samples used to detect differential expressions included prostate tissue (*n* = 25), urine (*n* = 12), whole blood (*n* = 7), and serum (*n* = 4). In seven studies, two types of biological samples among those mentioned above were analyzed.

The most frequently used technique for differential expression analysis was reverse transcription followed by real-time quantitative polymerase chain reaction (RT-qPCR, also referred to as RT-PCR or qPCR) (*n* = 55), followed by microarray (*n* = 1) and NanoString (*n* = 1), while one study did not report the technique used.

The statistical methods used to assess differential expression included Student’s *t*-test (*n* = 24), the Mann–Whitney U test (*n* = 12), ANOVA (*n* = 6), logistic regression models (*n* = 4), Wilcoxon test (*n* = 3), Kruskal–Wallis test (*n* = 3), chi-square test (*n* = 2), and ROC analysis (*n* = 1). The remaining three studies did not specify the statistical method used.

The newly identified ncRNA molecular diagnostic biomarkers comprised microRNAs (miRNAs), long non-coding RNAs (lncRNAs), circular RNAs (circRNAs), small nucleolar RNAs (snoRNAs), and PIWI-interacting RNAs (piRNAs), which were reported in 32, 19, 4, 1, and 1 studies, respectively. One study reported more than one type of RNA biomarker (small nuclear RNA [ARNsn] and miRNAs).

Overall, the methodological quality of the 58 included studies showed moderate to high variability, with compliance percentages ranging from 68.2% to 100.0%. The mean methodological quality score was 82.5% (SD = 9.0%), indicating that, on average, the included studies exhibited a moderate-to-high level of methodological rigor. Six studies achieved the maximum possible score, reflecting strong methodological robustness, particularly with respect to clarity of study design, outcome measurement, and reporting of results. In contrast, studies with lower quality scores, with compliance levels close to 68%, primarily showed methodological limitations related to insufficient sample size justification, limited control of confounding variables, and incomplete reporting of variance estimates (Supplementary File Sheet S14).

### 3.2. The miRNA-Based Molecular Diagnostic Biomarkers

A total of 94 miRNAs showing significant differential expression (*p* < 0.05) were identified across 32 studies focused exclusively on miRNAs and one study that analyzed both RNA types. The top five most significant miRNAs were miR-183-5p (*p* = 4.33 × 10^−10^), miR-205 (*p* = 9.10 × 10^−10^), miR-32-5p (*p* = 8.5 × 10^−8^), miR-187-3p (*p* = 4.03 × 10^−7^), and miR-9-5p (*p* = 1.2 × 10^−6^). In the case of fold change, the top five upregulated miRNAs (FC > 2) were miR-9-5p (FC = 8.8), miR-9-3p (FC = 6.29), miR-183-5p (FC = 6.2), miR-487b-3p (FC = 6.06), and miR-221-3p (FC = 5.47). Conversely, the top five downregulated miRNAs (FC < −2) were miR-181b-5p (FC = −19), miR-494-3p (FC = −5.22), miR-320a (FC = −4.2), miR-32-5p (FC = −4.11), and miR-3128 (FC = −4.06).

miR-145 was the most frequently reported miRNA, identified in five articles. It was followed by miR-141, miR-21-5p, and miR-32, each reported in four studies; miR-125b, miR-221, miR-16, and miR-375 were reported in three studies; while four miRNAs (miR-20a, miR-182-5p, miR-205, and miR-454) were reported in two articles. The remaining 59 miRNAs were mentioned in only one study (Supplementary File Sheet S6).

### 3.3. The lncRNA-Based Molecular Diagnostic Biomarkers

A total of eight lncRNA biomarkers showing significant differential expression (*p* < 0.05) were reported across 19 articles. All biomarkers were measured using RT-qPCR-based approaches. Among these, *MYU* (*p* = 0.0001) was detected in urine samples from 100 Chinese participants (59 cases and 41 controls); *MIR22HG* (*p* < 0.001) in tissue samples from 22 Chinese participants (13 cases and 9 controls); *FR0348383* (*p* ≤ 0.001) in urine samples from 213 Chinese participants (72 cases and 141 controls); *NEAT1* (*p* = 0.009, FC = 2.1) in plasma samples from 60 Romanian participants (37 cases and 23 controls); *PCAT-1* (*p* ≤ 0.03) in plasma samples from 80 Iranian participants (40 cases and 40 controls), *GAS5* (*p* = 0.05) in tissue samples from 25 Chinese participants (14 cases and 11 controls); *UCA1* (*p* = 0.05, FC = 18) in tissue samples from 10 Korean participants (10 tumor and 10 adjacent tissue) (Supplementary File Sheet S6).

Only *PCA3* was reported in more than one article (*n* = 12). The lncRNA *PCA3* was measured in urine, blood, and tissue, showing significant differential expression in all cases (*p* ≤ 0.05) and a fold change greater than 1 in the studies reporting it (*n* = 5). This up-regulation was consistently observed in prostate cancer cohorts from the United Kingdom, Italy, Brazil, the United States, Ukraine, Finland, Spain, and China, involving a total of 1357 participants (799 cases and 645 controls).

### 3.4. Other ncRNA-Based Molecular Diagnostic Biomarkers

Six studies reported circular RNAs (circRNAs), small nucleolar RNAs (snoRNAs), small nuclear RNAs (snRNAs), and PIWI-interacting RNAs (piRNAs). All biomarkers were validated in human samples using RT-qPCR-based methods and showed statistically significant differential expression (*p* < 0.05) (Supplementary File Sheet S6).

Among the circRNAs, circATXN10 (*p* = 0.0001), circSTIL (*p* = 0.003), circNFIA (*p* = 0.001; FC = 2.0), circZNF561 (*p* = 0.01; FC = −2.0), circABCC4 (*p* = 0.0001; FC = 2.31), circZNF577 (*p* = 0.037; FC = 1.56), circFAT3 (*p* = 0.0001; FC = −3.79), circITGA7 (*p* = 0.0001; FC = −2.96), and circATRNL1 (*p* = 0.0001; FC = −5.54) were identified across four studies, mainly using plasma and urine samples from Asian cohorts. In addition, the snoRNA SNORD78 (*p* < 0.0001) and its fragment sd78-3 (*p* = 0.0003) were found significantly up-regulated in prostate cancer tissue samples. One study also described the small nuclear RNA RNU1A-1 (*p* ≤ 0.030) as differentially expressed in tumor versus adjacent prostate tissue. In addition, one study reported four PIWI (piARN) as potential biomarkers, including piRNA002468 (*p* = 0.0068), piRNA349843 (*p* = 0.0151), piRNA382289 (*p* = 0.0084), and piRNA158533 (*p* = 0.0383), all of which were significantly up-regulated in prostate cancer tissue compared to controls (Supplementary File Sheet S6).

### 3.5. miRNA–mRNA Target Prediction

Of the 94 miRNAs identified across the primary studies, 66 had sufficient annotation to be included in the downstream in silico analysis, and 56 were successfully matched within the miRNA–target prediction platforms (Supplementary File Sheet S7). A total of 13,493 miRNA–mRNA interactions were predicted in common by miRDB, TargetScan v8.0, and miRTarBase v9.0, corresponding to 4916 unique target genes (Supplementary File, Sheet S8). In addition, 4669 interactions were commonly identified as prostate tissue–specific, yielding 2481 unique predicted targets (Supplementary File Sheet S9).

### 3.6. The miRNA–Protein Interaction Network

We conducted a miRNA–protein interaction network analysis using the protein-coding gene targets of the miRNAs reported as differentially expressed in patients with PCa. A total of 4916 + 2481 predicted targets were used as input for the STRING database. Non-protein-coding targets and non-connected nodes (after restricting the network to experimentally validated interactions with a confidence score ≥ 0.9) were removed from subsequent analyses. This filtering resulted in 1014 and 641 protein-target nodes in the unfiltered analysis (Supplementary File Sheet S8) and the prostate tissue–specific analysis (Supplementary File, Sheet S9), respectively, yielding 1311 unique protein targets in total. These unique proteins were subsequently re-entered into STRING to obtain the final interaction data (Supplementary File Sheet S10).

The resulting protein interaction network contained 1051 nodes and 1676 edges, comprising a main connected component with 738 nodes and 1398 edges, along with several smaller disconnected sub-networks. For downstream analyses, only the nodes belonging to the main connected component were retained (Supplementary File, Sheet S10). The information for the miRNAs targeting the proteins included in the network was retrieved, and a miRNA–protein interaction network was subsequently generated.

**Figure 2:**
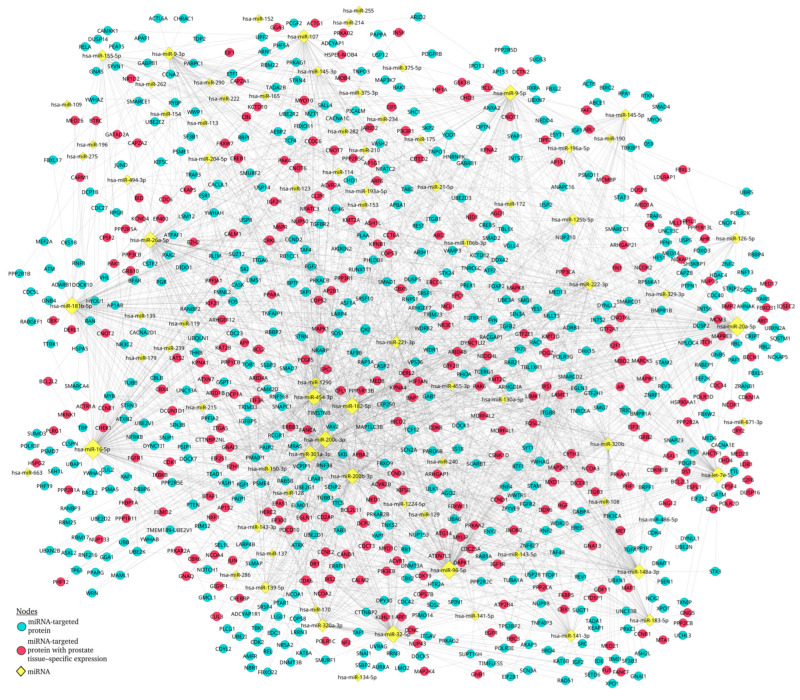
miRNA–protein interaction network. The network is displayed by Cytoscape and contains 845 nodes and 2235 edges. The yellow diamonds represent the miRNAs, the blue circles denote the predicted protein targets, and the red circles correspond to the protein targets with prostate tissue–specific expression. The size of the nodes is proportional to the number of interactions (degree).

The miRNA–protein interaction network comprised 845 nodes and 2335 edges (Figure 2 and Supplementary File Sheets S11 and S12). The most highly connected miRNA in the network was hsa-miR-16-5p (142 edges), followed by hsa-miR-20a-5p, hsa-miR-96-5p, hsa-miR-181b-5p, hsa-miR-32-5p, hsa-miR-182-5p, hsa-miR-454-3p, and hsa-miR-200c-3p, all of which displayed more than 100 edges.

Among protein nodes, QKI, YOD1, and TBL1XR1 were the most connected, with 19, 15, and 14 edges, respectively (see Table S1, which ranks potential biomarkers and their impacts in prostate cancer). Within the prostate-associated protein subset, CDK6 was the most connected node (14 edges), followed by ACVR2B, KMT2A, and PIK3R1, each with 13 edges (see Table S2, which ranks potential biomarkers and their impacts in prostate cancer).

### 3.7. Enriched Biological Networks

**Figure 3:**
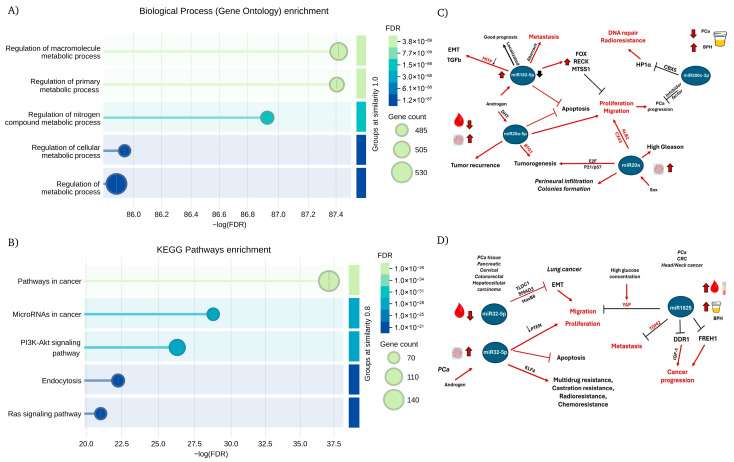
Functional enrichment results of miRNA-associated protein targets. (**A**) Top five Gene Ontology (GO) terms, ranked according to the most significant false discovery rate (FDR) values. (**B**) Top five KEGG Orthology (KO) pathways, ranked according to the most significant FDR values. Node size is proportional to the number of significantly enriched genes within each group. (**C**) Differences in the enriched pathways associated with up-regulated versus down-regulated miR20a, miR182-5p and miR200c-3p. (**D**) Differential regulation by miR-32-5p and miR1825 in blood, tissue and urine samples. Red arrows indicate induction of pro-tumorigenic routes, whereas black lines indicate inhibitory effects, suggesting pathways or molecules that are suppressed. Color-coded molecule labels distinguish inhibited nodes (red) from direct miRNA targets (black).

A total of 1311 unique protein targets were submitted to the STRING database for network enrichment analysis, yielding a highly significant PPI enrichment *p*-value < 1.0 × 10^−16^ (Supplementary File Sheet S13). A total of 2342 Gene Ontology (GO) terms were significantly enriched (FDR < 0.05), including 1808 Biological Process, 311 Cellular Component, and 223 Molecular Function terms. Among these, metabolic process–related GO categories were the most prominently enriched (Figure 3A). In the KEGG pathway analysis, 174 KO terms were significantly up-regulated (FDR < 0.05), with miRNA in cancer and cancer-associated pathways ranked among the top five enriched categories (Figure 3B).

## 4. Discussion

This study provides an integrated overview of non-coding RNAs proposed as diagnostic biomarkers for prostate cancer, combining evidence mapping with an in silico analysis of the miRNA regulatory landscape. From 58 primary studies, we identified 94 differentially expressed miRNAs, representing the most extensively studied ncRNA class, along with eight lncRNAs, of which *PCA3* showed the greatest consistency across sample types. Other ncRNA categories remain less explored. By integrating validated miRNAs with their predicted targets, we identified the miRNAs with the strongest influence within biological networks and highlighted key protein targets that may represent promising candidates for future clinical biomarker studies.

Most studies originated from Asia and Europe, which means the current ncRNA biomarker landscape does not fully reflect the global molecular profile of prostate cancer. This geographic concentration may influence the patterns observed, since population-specific genetic and environmental factors can affect ncRNA expression [79]. Regions such as Africa and South America remain underrepresented, underscoring the need for broader sampling to develop diagnostic signatures that are applicable across diverse populations. Most studies also included small sample sizes, which restricts the generalization of the reported ncRNA biomarkers. Furthermore, only a few articles reported diagnostic metrics such as specificity, sensitivity, or AUC values, making it difficult to assess the true clinical performance of these candidates. Larger and better standardized studies are needed to confirm their diagnostic value. While the methodological quality of individual studies was generally moderate to high according to QualSyst, substantial inter-study heterogeneity was observed in terms of analytical platforms, normalization strategies, biological sample types (tissue, urine, serum, whole blood), and cohort composition. This heterogeneity has important implications for biomarker discovery, as ncRNA expression levels and even the direction of differential expression may vary depending on the biological matrix analyzed, the cellular composition of the sample, and the technical workflow applied [80,81]. Consequently, direct quantitative comparisons across studies are limited, and inconsistencies reported for certain ncRNAs likely reflect methodological and biological context rather than true biological contradiction.

To address this challenge, we deliberately adopted an evidence-mapping strategy focused exclusively on ncRNAs whose differential expressions were validated in patient-derived samples, excluding discovery-only or in silico-based reports without clinical confirmation. Rather than attempting to identify universally concordant expression patterns across heterogeneous datasets, our approach emphasizes the integration of clinically validated biomarkers into a network-based framework. This allows the identification of regulatory hubs and convergent molecular pathways that remain biologically relevant despite inter-study variability. The eight miRNAs identified as central nodes in the interaction network exhibited high regulatory connectivity, suggesting that they may play key roles within the modeled molecular landscape of prostate cancer. Rather than implying direct clinical applicability, this network centrality highlights their potential relevance as regulatory hubs and prioritization candidates for future functional and clinical validation studies. In this context, network-based centrality should be interpreted as a hypothesis-generating metric that helps identify ncRNAs involved in convergent oncogenic pathways, rather than as evidence of diagnostic readiness.

The most connected node in the network was miR-16, validated in three different populations. In South African patients (24 PCa and 10 BPH), the miR-194-5p/miR-16-5p ratio correlated with disease severity and was proposed as a diagnostic biomarker [32]. A small U.S. study also found significant differences in miR-16 expression between normal and PCa epithelium (*p* < 0.05), and between normal epithelium and stromal tissue (*p* < 0.01), miR-16 was down-regulated in PCa epithelium and up-regulated in stroma [46]. In German serum samples from 55 patients, miR-16 was upregulated in PCa and decreased significantly after radical prostatectomy [47]. Despite these findings, miR-16 is still commonly used as an endogenous qPCR normalizer [82], even though PCa cell studies show marked down-regulation of miR-16-5p and functional targeting of *AR* and *EGFR*, both closely linked to prostate cancer biology [83].

In addition, hsa-miR-20a-5p was the second most connected node in the network. Its validation has been reported in two independent studies. In a U.S. cohort, hsa-miR-20a-5p was down-regulated in blood samples from 28 prostate cancer patients compared to 12 healthy controls [30]. In contrast, a study from Iran found increased serum levels of hsa-miR-20a-5p in 40 PCa patients compared to 40 non-cancerous volunteers, with a significant reduction after surgery [36]. These findings suggest that the direction of change may depend on sample type and clinical context of the studied population. Finally, the third most connected node was hsa-miR-96-5p, which has been validated in tissue samples in a single study from Poland involving 23 prostate cancer and 22 benign prostatic hyperplasia cases. In this cohort, hsa-miR-96-5p was down-regulated in PCa and showed a negative correlation with Gleason score [66].

An important observation is that the miRNAs most frequently reported in clinical studies did not necessarily correspond to the most influential nodes in the interaction network. Of the 12 miRNAs validated in more than one study, only four (miR-16, miR-20a, miR-182-5p, and miR-454) showed more than 100 network connections. The remaining four highly connected hubs appeared in only a single study. This mismatch shows that clinical replication and network centrality represent different dimensions of biomarker relevance and highlights the need to integrate both types of evidence when prioritizing miRNAs for future diagnostic validation.

In the proteins predicted as miRNA targets, the most highly connected prostate-associated protein in the network was CDK6, a catalytic subunit of the cyclin-dependent kinase complex that regulates G1 phase progression and the G1/S cell cycle transition. Altered expression of CDK6 has been reported in several human cancers [84]. In our interaction network, CDK6 was targeted by 14 miRNAs validated in prostate cancer, including the network hub hsa-miR-16-5p. Other highly connected proteins included ACVR2B (13 edges) and CRKL (12 edges), which were linked to most of the miRNA hubs, but currently have limited evidence supporting a specific role in prostate cancer. In the case of the top ten non-prostate-specific protein targets with the highest connectivity in the network, ITGB8, YOD1, and RNF38 were also identified as targets of several miRNA hubs. Although these proteins have limited evidence linking them to prostate cancer (Table S1), their strong network connectivity suggests that they may represent novel candidates for future investigation.

Regarding the results of the network enrichment analysis, the predicted protein targets of the validated miRNAs showed significant enrichment in pathways such as miRNAs in cancer, PI3K–Akt signaling, and androgen-related pathways. These pathways are central to prostate tumorigenesis [85]. The PI3K–Akt axis and androgen receptor signaling interact extensively during prostate cancer progression, influencing cell proliferation, survival, and metabolic adaptation. Several of the miRNA hubs identified in our network have previously been associated with these regulatory circuits. Overall, these findings indicate that the most influential miRNAs converge on biological processes known to promote tumor growth and metabolic reprogramming in prostate cancer, reinforcing their potential relevance as diagnostic biomarkers.

In this way, the functional implications of these miRNA hubs are further illustrated in Figure 3C,D. These diagrams summarize the pathway-level effects of the most influential miRNAs identified in the network, showing whether their activity leads to pathway activation or inhibition. Together, these visual maps highlight how miR-20a-5p [86], miR-1825p [21,87,88,89,90], miR-182-5p [91,92], miR-200c-3p [23,93,94], and miR-32-5p [30,95,96,97] converge on key oncogenic circuits including proliferation, migration, apoptosis resistance, EMT, and therapy resistance, by modulating targets such as PTEN, E2F, FOX, RECK, and DDR1, complementing the enrichment analysis and reinforcing their potential biological relevance in PCa.

Several authors have reported both proto-oncogenic and tumor-suppressive activities for different miRNAs, underscoring their dual roles in different cancers, whereas their concentration and localization (blood or tissue) could induce a differential response not only as indicators of disease severity but also as candidates for identifying therapeutic vulnerabilities that may help guide more precise diagnostic and therapeutic strategies [85,86,87,88,89,90,91,92,93,94,95,96,97]. Overall, the visual maps complement the enrichment analysis by revealing how these miRNA hubs orchestrate key molecular circuits involved in tumor progression, recurrence, and therapeutic resistance, highlighting their potential as both mechanistic indicators and targets for precision-based interventions in prostate cancer.

Clinically, our findings support the development of multimarker diagnostic panels that integrate the most influential miRNAs with established indicators such as PSA to improve specificity and reduce unnecessary biopsies. The convergence of miRNA hubs on key prostate cancer pathways also highlights the potential of biologically informed panels over single biomarkers. To advance these candidates toward clinical implementation, future research should rely on large, multicenter and multiethnic cohorts and apply standardized analytical and reporting frameworks. Such efforts will be crucial to validate the robustness and generalization of ncRNA-based diagnostics.

This study has several limitations. The in silico analyses depend on database annotations and prediction algorithms, so the identified interactions require experimental confirmation. We did not perform in vivo or in vitro validation, which limits the direct biological verification of the predicted targets. Experimental validation constitutes a necessary next step for future research. In addition, the primary studies included showed considerable variability in analytical platforms, sample processing, and normalization methods, which may introduce heterogeneity and batch effects. These factors, together with the generally small sample sizes, reduce comparability across studies and highlight the need for standardized approaches in future research.

## 5. Conclusions

This study provides a comprehensive integration of clinical evidence and in silico analyses to characterize non-coding RNAs associated with prostate cancer diagnosis. By synthesizing data from 58 primary studies, we identified 94 validated miRNAs, eight lncRNAs, and other minority additional ncRNA classes. Although none of the candidate ncRNA biomarkers demonstrate sufficient reproducibility and robustness for clinical diagnostic implementation, the network-based analysis revealed eight miRNAs with high regulatory centrality and identified key protein targets and enriched pathways that align with known mechanisms of prostate tumorigenesis. Together, these exploratory results help refine the list of promising biomarker candidates and highlight potential new molecules and pathways for investigation. Future work should focus on prospective validation studies of these candidates, and on evaluating their performance in biologically informed multimarker panels that complement existing diagnostic tools such as PSA.

## Data Availability

The original contributions presented in this study are included in the article/Supplementary Materials. Further inquiries can be directed to the corresponding author.

## Supplementary Materials

The following supporting information can be downloaded at: https://www.mdpi.com/article/10.3390/life16010095/s1. Figure S1: Protein–Protein Interaction Network derived from miRNA predicted target genes; Table S1. The top ten connected proteins in the network ordered by degree [98,99,100,101,102,103,104,105,106,107]; Table S2. The top ten highly connected prostate-associated proteins in the network ordered by degree [108,109,110,111,112,113,114,115,116,117]; Supplementary File S1.xlsx; ncRNA_Prostate.cys.

## Abbreviations

The following abbreviations are used in this manuscript:

ACVR2B	Activin A receptor type IIB
ANOVA	Analysis of Variance
AUC	Area Under the Curve
BPH	Benign Prostatic Hyperplasia
CDK6	Cyclin-Dependent Kinase 6
circRNA	Circular RNA
DDR1	Discoidin Domain Receptor 1
EMT	Epithelial–Mesenchymal Transition
FG	Fold Change
FDR	False Discovery Rate
FOX	Forkhead Box Transcription Factors
GO	Gene Ontology
KEGG	Kyoto Encyclopedia of Genes and Genomes
lncRNA	Long Non-Coding RNA
miRNA	microRNA
ncRNA	Non-Coding RNA
PCa	Prostate Cancer
*PCA3*	Prostate Cancer Antigen 3
PI3K	Phosphoinositide 3-Kinase
piRNA	PIWI-Interacting RNA
PRISMA	Preferred Reporting Items for Systematic Reviews and Meta-Analyses
PSA	Prostate-Specific Antigen
PTEN	Phosphatase and Tensin Homolog
qPCR	Quantitative Polymerase Chain Reaction
RT-qPCR	Reverse Transcription Quantitative Polymerase Chain Reaction
QKI	Quaking Homolog
RNU1A-1	RNA, U1 Small Nuclear 1A
ROC	Receiver Operating Characteristic
snoRNA	Small Nucleolar RNA
snRNA	Small Nuclear RNA
STRING	Search Tool for the Retrieval of Interacting Genes/Proteins
TBL1XR1	Transducin Beta-Like 1 X-Linked Receptor 1
UCA1	Urothelial Cancer Associated 1
