# Supplementary material for: Non-Coding RNA Biomarkers in Prostate Cancer: Evidence Mapping and In Silico Characterization
Source: Life (Basel). 2026 Jan 8;16(1):95. doi: 10.3390/life16010095 (PMC12842983; doi:10.3390/life16010095)

Figure S1. Protein–Protein Interaction Network derived from miRNA predicted target genes. The network is displayed by Cytoscape and contains 738 nodes and 1398 edges. The blue circles denote the predicted protein targets, and the red circles correspond to the protein targets with prostate tissue–specific expression. The size of the nodes is proportional to the number of interactions (degree).

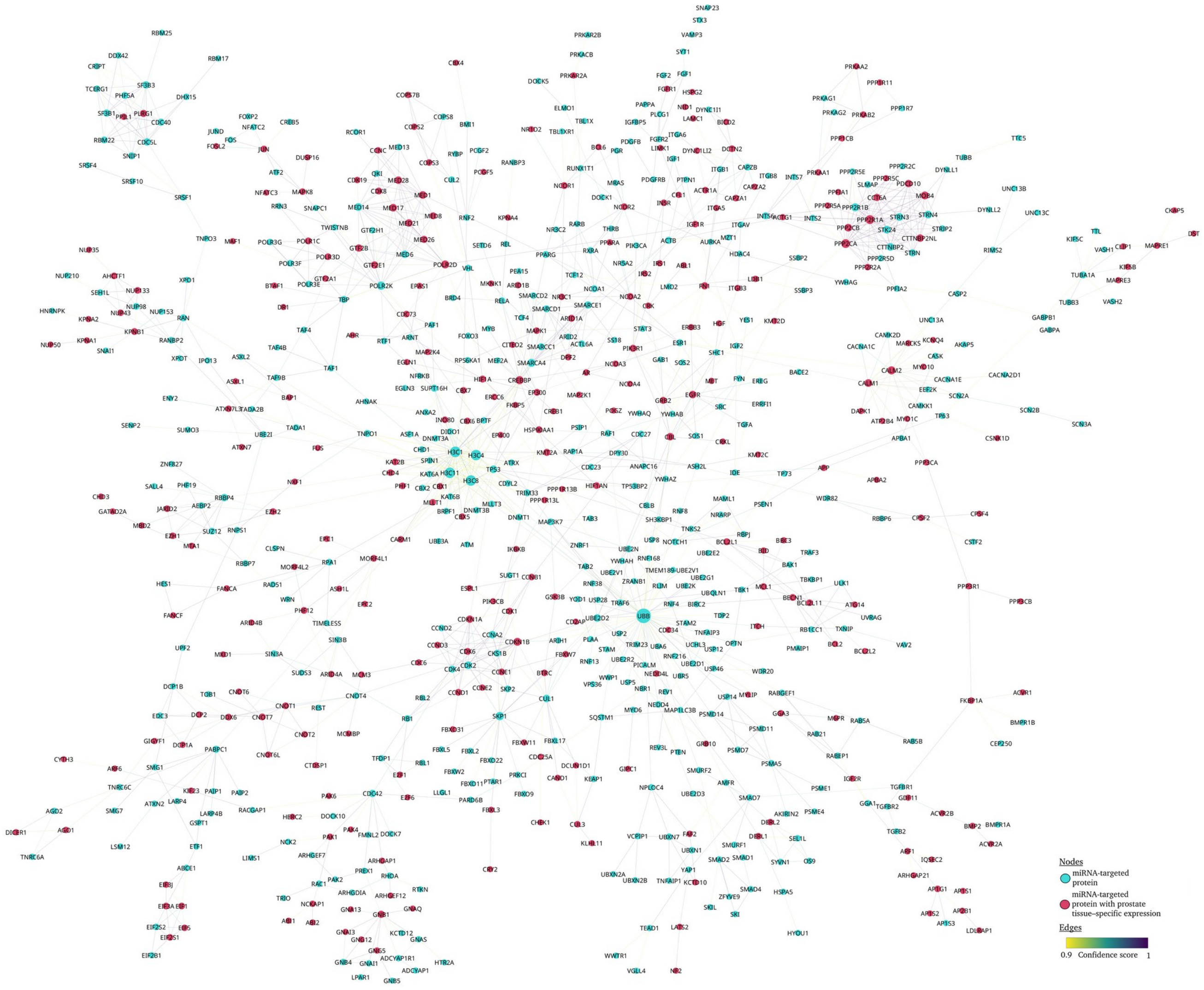

Supplement: Supplementary file 1 [file life-16-00095-s001.zip › Supplementary_Figure_S1.pdf]
