# Supplementary material for: Non-Coding RNA Biomarkers in Prostate Cancer: Evidence Mapping and In Silico Characterization
Source: Life (Basel). 2026 Jan 8;16(1):95. doi: 10.3390/life16010095 (PMC12842983; doi:10.3390/life16010095)
Supplement: Supplementary file 1 [file life-16-00095-s001.zip › Supplementary_Table_S1.pdf]

Table S1. The top ten connected proteins in the network ordered by degree

Protein	Degree	N° of articles related with cancer	N° of articles related with prostate cancer	Description
QKI	19	156	11	QKI functions as an RNA-binding protein that promotes the biogenesis of circAKT3 in prostate cancer. By facilitating AKT3 circularization through flanking Alu elements, QKI increases circAKT3 levels, which in turn enhances tumor cell proliferation, migration, and metastasis [98].
YOD1	15	19	2	YOD1 is directly associated with resistance to Enzalutamide (ENZ), an androgen receptor antagonist used for metastatic castration-resistant prostate cancer (mCRPC) [99].
TBL1XR1	14	122	13	TBL1XR1 is overexpressed in prostate cancer cells, and its suppression, via targeting by the miR-199a/214 cluster reduces cell proliferation, induces apoptosis, causes cell-cycle arrest, and significantly increases the sensitivity of prostate cancer cells to the anti-EGFR antibody Nimotuzumab [100].
ITGB8	13	78	1	The high level of expression of both EPHB4 and ITGB8 in clinical prostatic intraepithelial neoplasia samples suggests that their increased expression is an early event in the development of prostate cancer [101].
TNPO1	13	2091	7	In PCa, TNPO1 is implicated through the discovery of the TNPO1–IKBKB gene fusion, a novel rearrangement that drives marked overexpression of IKK-β and aberrant NF-κB pathway activation. This fusion places IKBKB under TNPO1's regulatory control, promoting oncogenic signaling associated with tumor progression and aggressive disease biology [102].
PTEN	12	16910	545	PTEN loss promotes prostate cancer aggressiveness through PI3K–AKT hyperactivation and serves as a major prognostic and biological driver of disease progression [103].
RNF38	11	19	1	High level of RNF38 mRNA was observed in prostate carcinoma compared with normal tissue [104].
YWHAG	11	86	1	YWHAH in the European-American population were found to be associated with aggressive PCa using at least one model [105].
ESR1	9	4832	202	ESR1 (Estrogen Receptor 1): involved in susceptibility to prostate cancer (PCa) by stimulating abnormal prostate growth and controlling cell proliferation/death. Polymorphisms in ESR1 can affect its transcriptional activity:<br>PvuII (C.T): Significantly associated with an increased risk of PCa in Asian populations. Increased Risk (OR): OR=2.27 (homozygous TT vs CC model, in Indian population). P-value: $P<0.001$.<br>XbaI (A.G): May increase the risk of PCa in the US population. Increased Risk (OR): OR=1.14 (G allele model vs A). P-value: $P=0.045$ [106].

STRN	9	117	2	STRN4 (Striatin-4): promueve la proliferación celular e inhibe la apoptosis en la próstata. Se verificó una sobreexpresión significativa del ARNm de STRN4 en muestras cancerosas, con un aumento medio de 1,81 veces y un aumento máximo de 6,05 veces, P < 0,001 [107].
