# Supplementary material for: Non-Coding RNA Biomarkers in Prostate Cancer: Evidence Mapping and In Silico Characterization
Source: Life (Basel). 2026 Jan 8;16(1):95. doi: 10.3390/life16010095 (PMC12842983; doi:10.3390/life16010095)
Supplement: Supplementary file 1 [file life-16-00095-s001.zip › Supplementary_Table_S2.pdf]

Table S2. The top ten highly connected prostate-associated proteins in the network ordered by degree.

Protein	Degree	N° of articles related with cancer	N° of articles related with prostate cancer	Description
CDK6	14	2663	125	CDK6 is markedly upregulated in enzalutamide-resistant castration-resistant prostate cancer (EnzR CRPC) in both cell lines and clinical samples [108].
ACVR2B	13	83	5	ACVR2B acts as a key mediator through which prostate-tumor–derived GDF11 drives severe muscle loss during androgen-deprivation therapy [109].
KMT2A	13	1998	24	KMT2A upregulated in ERG+ PCa cells/tumors; drives ERG tumor initiation via ETS/STAT3 chromatin opening (scATAC-seq); knockout impairs organoid/tumor growth (ANOVA $p<0.05$) [110].
PIK3R1	13	464	30	PIK3R1 markedly downregulated in prostate cancer, with strongly significant reductions reported across independent cohorts ($p = 4.99\times10^{-5}$ and $p = 5.87\times10^{-7}$) [111].
CRKL	12	417	3	CRKL: upregulated in advanced PCa tumors; functions as a strong AR coactivator that interacts with the AR complex and recruits to the KLK2 enhancer region; Overexpression enhances AR transcriptional activity up to 3.9 fold (mediado por el dominio SH3-C), while its knockdown reduces AR activity by 50%. Expression of CRKL combined with growth factor stimulation (PDGF/EGF) significantly rescues AR transcriptional activity, bypassing casodex inhibition ($p\leq0.03$), by utilizing an alternative growth factor signaling pathway to drive androgen-independent signaling [112].
DICER1	12	1286	24	DICER1 (miR processing enzyme) positively correlated with prostatic 1,25(OH)2D metabolite levels in the stroma of PCa patients (RT-qPCR, p-trend demonstrated across tertiles). A high epithelial:stromal ratio of DICER1 protein levels was significantly associated with future biochemical recurrence (BCR). The highest quartile of the epi/stroma DICER1 ratio predicted BCR with an Odds Ratio (OR) of 3.068 ($p=0.04$). The regulation of miR expression (e.g., miR-126-3p and miR-154-5p were upregulated dose-dependently, p trend ≤0.0301) and

				DICER1 in the stroma by 1,25(OH)2D may provide protection against aggressive PCa. The regulation of miRs was VDR-dependent [113].
KPNA1	11	44	2	KPNA1 (Importin alpha 5): confirmed propensity to act as a target in PC; analysis confirmed the apparent propensity of mRNAs encoded by KPNA1 to act as targets for microRNAs associated with PC (18 PC-associated SNPs were included in the analysis). This pathway suggests that the genetic and molecular pathology of the nuclear import pathway (of which KPNA1 is a major component) is an important potential target in the pathogenesis of PC. Analysis of PC SNP-guided microRNA maps confirmed the propensity to target the mRNA encoded by KPNA1. Regulation/Values: No specific fold change values or p-values quantifying the up- or downregulation of KPNA1 expression in prostate cancer tissue were provided within the sources [114].
AGO1	9	153	9	hAGO1 (Argonaute 1): overexpressed in PC3 prostate cancer cells/tumors; functions as a nuclear regulator that represses the transcription of oncogenic pathways and enhances the recruitment of RNA polymerase II (RNAPII) to active promoters. Its depletion induces cell cycle arrest at G0/G1 in PC3 cells. Furthermore, mutations affecting the dosage and function of hAGO1 are significantly correlated with tumor development, metastasis, and survival in prostate cancer patients [115].
ASH1L	9	67	3	ASH1L (Histone methyltransferase) is genetically amplified and overexpressed in metastatic prostate cancer (PCa) (occurs in >40% of metastatic PCa); it functions as an epigenetic driver that cooperates with HIF-1α to induce a pro-metastatic transcriptome through methylation of histones H3K4me3 and H3K36me3.<br>ASH1L/HIF-1α regulatory axis promotes IGF-2 expression, which induces monocyte differentiation into LA-TAMs (Lipid-Associated Macrophages) and maintains their pro-tumor phenotype by enhancing oxidative phosphorylation (OXPHOS). Depletion/Knockout of ASH1L markedly suppresses PCa cell invasion (ANOVA $p<0.05$ for the 3D invasion assay) and bone metastases; Overexpression promotes metastatic outgrowth in bone [116].

CCDC6	9	424	3	CCDC6 (tumor suppressor protein): expresses appreciable levels in prostate cancer (PCa) cell lines and detectable levels in 68% of the primary prostate tumors analyzed. Its stability and turnover are regulated by the deubiquitinase USP7. CCDC6 expression was significantly correlated with USP7 levels in primary prostate tumors (correlation coefficient $R = 0.855$, $p \leq 0.05$ in rank correlation analysis). Pharmacological inhibition of USP7 accelerates CCDC6 degradation, impairing Homologous Recombination (HR)-directed DNA repair and reducing Rad51 foci after irradiation. Reduction of CCDC6 sensitizes PCa cells (including castration-resistant cells such as 22Rv1) to treatment with the PARP inhibitor Olaparib [117].
